# Long-Term Outcomes of Switching from Fixed-Dose to As-Needed Regimen for Treating Submacular Hemorrhage Secondary to Polypoidal Choroidal Vasculopathy

**DOI:** 10.3390/jcm9082637

**Published:** 2020-08-14

**Authors:** Jae Hui Kim, Jong Woo Kim, Chul Gu Kim, Dong Won Lee

**Affiliations:** Department of Ophthalmology, Kim’s Eye Hospital, Konyang University College of Medicine, Seoul 150-034, Korea; kjwood@kimeye.com (J.W.K.); chulgukim@kimeye.com (C.G.K.); mediceye@kimeye.com (D.W.L.)

**Keywords:** polypoidal choroidal vasculopathy, hemorrhage, fixed-dose regimen, as-needed regimen, anti-vascular endothelial growth factor, reactivation, recurrence

## Abstract

Background: The aim of this study was to evaluate outcomes in patients with submacular hemorrhage secondary to polypoidal choroidal vasculopathy (PCV) after switching treatment from a fixed-dose to an as-needed regimen. Methods: This retrospective study included 19 patients with submacular hemorrhage secondary to PCV who were treated with fixed-dose intravitreal aflibercept during the first 56 weeks. After 56 weeks, the treatment regimen was switched to an as-needed regimen. The incidence and timing of lesion reactivation during the as-needed phase were evaluated. The best-corrected visual acuity (BCVA) at baseline (beginning of the regimen) and the final follow-up were compared. Multivariate analysis was performed to determine the factors associated with lesion reactivation. Results: During the mean follow-up period of 27 ± 7.3 months, lesion reactivation was noted in 10 patients (52.6%; mean time period: 12.2 ± 9.1 months) in the as-needed phase. Reactivations were treated with anti-vascular endothelial growth factor (VEGF) injections (mean, 4.1 ± 2.6). The mean logarithm of the minimum angle of resolution (logMAR) BCVA was 0.26 ± 0.34 at baseline and 0.31 ± 0.38 at final follow-up (*p* = 0.212). Deterioration of ≥0.2 logMAR BCVA was noted in two patients (10.5%). In multivariate analysis, large lesion size was closely associated with a high risk of lesion reactivation (*p* = 0.009). Conclusion: Visual acuity was relatively stable after switching from a fixed-dose to an as-needed regimen, with no definite visual deterioration in the majority of patients. We conclude that patients with large lesions should be carefully monitored when switching to an as-needed regimen.

## 1. Introduction

Polypoidal choroidal vasculopathy (PCV), also called aneurysmal type 1 neovascularization, is a distinct type of choroidal neovascularization that is prevalent in the Asian population [1,2,3,4].

Previously, photodynamic therapy (PDT) was the mainstay of treatment for PCV [5]. However, with the advent of anti-vascular endothelial growth factor (VEGF) therapy, the treatment pattern has changed substantially [6,7]. Currently, several treatment methods, including direct photocoagulation, PDT monotherapy, anti-VEGF monotherapy, and the combination of PDT and anti-VEGF therapy are used to treat PCV [7]. Although anti-VEGF monotherapy is a simple and effective method, PDT and direct photocoagulation still have a valid role in treatment [6,7]. Koh et al. have suggested direct photocoagulation as an initial treatment for extrafoveal PCV [6]. In subfoveal and juxtafoveal PCV, PDT alone or in combination with anti-VEGF therapy has been suggested as an initial treatment. Anti-VEGF monotherapy can then be performed after the complete regression of polyps. However, retreatment with PDT has been suggested when the polyps do not regress completely [6]. When compared to anti-VEGF monotherapy, the combination of PDT and anti-VEGF therapy shows a superior outcome compared with anti-VEGF monotherapy, with the added benefit of fewer anti-VEGF injections [8,9].

A high risk of submacular hemorrhage development is a characteristic of PCV [10]. Investigators have attempted to develop an effective treatment method for hemorrhage [11] because it may result in diffuse degeneration of the outer retinal layers and the retinal pigment epithelium [12,13]. Intravitreal administration of anti-VEGF is one of the effective treatment modalities for submacular hemorrhage secondary to PCV [14,15]. Studies have reported anatomical and functional improvements in both the as-needed regimen [14,16,17] as well as the fixed-dose regimen [15]. It is difficult to anticipate the complete cure of PCV; therefore, long-term treatment is usually required to preserve vision [18]. In clinical practice, treatment regimens may be switched at the discretion of the treating doctors during this long-term treatment.

A shortcoming of a fixed-dose or treat-and-extend regimen is the necessity for frequent injections [19,20]. Considering the high cost of anti-VEGF drugs, this regimen may increase a patient’s financial burden. Thus, if the disease is in a stable state, switching to the more efficient as-needed regimen can be considered a treatment option [21,22]. Hence, information regarding outcomes after switching to an as-needed regimen may aid clinicians in establishing the future management plan. To date, none of the studies have evaluated this topic in PCV.

We recently reported 56-week treatment outcomes of bimonthly fixed-dose intravitreal aflibercept therapy for treating submacular hemorrhage secondary to PCV and neovascular age-related macular degeneration (AMD) [15]. In that study, patients were administered eight injections during the 56-week study period. Consequently, in the Early Treatment Diabetic Retinopathy Study, best-corrected visual acuity (BCVA) markedly improved from 52.9 ± 17.8 letters at baseline to 71.8 ± 16.1 letters at week 56, with complete resolution of submacular hemorrhage. After 56 weeks, the treatment regimen was switched to an as-needed regimen in all patients. In the present study, we evaluated the long-term outcomes after switching to an as-needed regimen in PCV patients.

## 2. Materials and Methods

The present retrospective study was performed at a single center. The study was approved by the Institutional Review Board of Kim’s Eye Hospital, and was conducted in accordance with the tenets of the Declaration of Helsinki.

### 2.1. Patients

In our previous clinical trial, we included patients diagnosed with fovea-involving subretinal hemorrhage, with >1-disc diameter area, secondary to neovascular AMD and PCV [15]. Patients with PCV who were followed up for at least 12 months after clinical trial completion were included in the present study.

### 2.2. Examinations, Treatment, and Follow-Up

In the previous clinical trial [15], patients were initially administered three injections of aflibercept (2 mg/0.05 mL; Eylea^®^; Regeneron Pharmaceuticals, Tarrytown, NY, USA) every month, followed by a fixed-dose injection every 8 weeks. A total of eight injections (weeks 0, 4, 8, 16, 24, 32, 40, and 48) were administered during the 56-week study period. At the final follow-up (56 weeks), the following examinations were performed: (1) BCVA measurement; (2) fundus photography; (3) fluorescein angiography; (4) indocyanine green angiography (ICGA); and (5) optical coherence tomography (OCT). However, aflibercept injection was not administered at this final follow-up visit. All the OCT images were obtained using the Spectralis^®^ device (Heidelberg Engineering GmbH, Heidelberg, Germany).

After the 56-week follow-up, the treatment regimen was changed to an as-needed one. Additional injections were administered only if there was evidence of reactivation, such as an increase in intraretinal/subretinal fluid or development of hemorrhage. During the first 12 months, the patients were followed up every 1 to 2 months. Then, the interval between follow-ups was increased to 3 to 4 months for those showing no reactivation of the lesion. Retreatment was performed with either aflibercept or bevacizumab (1.25 mg/0.05 mL; Avastin^®^, Genentech, San Francisco, CA, USA).

### 2.3. Outcome Measures

The final follow-up (week 56 visit) of the previous clinical trial was the baseline time point of the present study. The patients’ detailed clinical course, including the incidence and timing of lesion reactivation and recurrent hemorrhage, was documented. The BCVA and central retinal thickness (CRT) measured at baseline were compared with those measured at 12 months and at the final follow-up. Patients were divided into the following two groups according to lesion size at baseline: <3.0 mm^2^ (small lesion size group) and ≥3.0 mm^2^ (large lesion size group). The BCVAs measured at the three time points were compared within each group. If the patient did not visit exactly after 12 months, values from the closest visit were used for analysis. To determine factors associated with lesion reactivation, multivariate analysis was performed using the following variables: age (<60 years vs. ≥60 years); sex (male vs. female); BCVA (<0.5 logarithms of the minimum angle of resolution (logMAR) vs. ≥0.5 logMAR); CRT (<250 µm vs. ≥250 µm); number of polypoidal lesions at baseline (<3 vs. ≥3); lesion size at baseline (<3.0 mm^2^ vs. ≥3.0 mm^2^); and serous pigment epithelial detachment (PED). CRT was defined as a mean retinal thickness of 1 mm diameter positioned at the center of the fovea. The lesion size was defined as the area of the entire neovascular complex, including branching vascular networks and polypoidal lesions. ICGA images were analyzed using Heidelberg Eye Explorer^®^ software (Heidelberg Engineering GmbH) to measure the lesion size.

### 2.4. Statistical Analyses

Data are presented as means ± standard deviations or numbers (percentages) as applicable. All statistical analyses were performed using SPSS software (version 12.0 for Windows; IBM Corporation, Armonk, NY, USA). BCVA values were converted to logMAR values for analysis. Values between different time points were compared using the Wilcoxon signed-rank test with Bonferroni’s correction. Multivariate analysis was performed using a binary logistic regression model. *p*-Values < 0.05 were considered statistically significant.

## 3. Results

Twenty-two of the twenty-nine patients included in the previous clinical trial were diagnosed with PCV, among whom nineteen were followed up for 12 months or longer after completion of the clinical trial. Consequently, we analyzed the results of these 19 patients. Patients’ baseline characteristics are summarized in Table 1.

**Figure 1:**
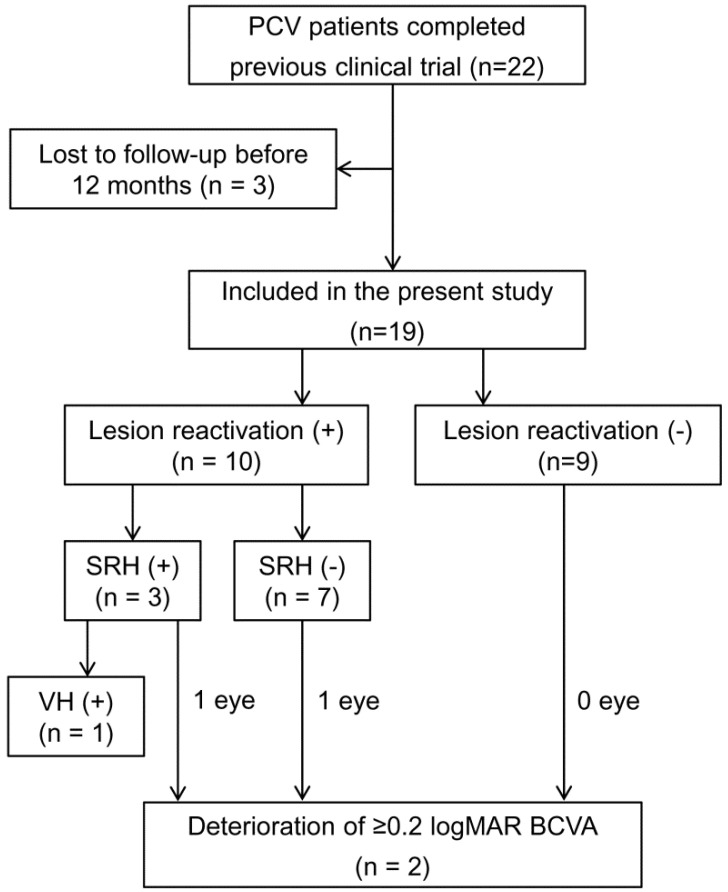
Clinical course in the included eyes. BCVA = best-corrected visual acuity; logMAR = logarithm of minimum angle of resolution; PCV = polypoidal choroidal vasculopathy, SRH = subretinal hemorrhage, VH = vitreous hemorrhage. The baseline was 56 weeks after fixed-dose therapy.

During the mean follow-up period of 27 ± 7.3 months, lesion reactivation was noted in 10 patients (52.6%) at a mean time of 12.2 ± 9.1 months (14.4 ± 9.1 months after the last aflibercept injection), 3 of whom also showed hemorrhage development. These 10 patients were followed-up for a mean of 15.3 ± 9.5 months after the first reactivation. During follow-up, anti-VEGF injections (mean, 4.1 ± 2.6) were administered as follows: seven patients received aflibercept alone; one patient, bevacizumab alone; and two patients, both aflibercept and bevacizumab. One patient underwent vitrectomy for vitreous hemorrhage followed by cataract surgery due to cataract progression after vitrectomy. In another patient, cataract surgery was performed during the follow-up period. Figure 1 presents the detailed clinical course of all the patients.

**Figure 2:**
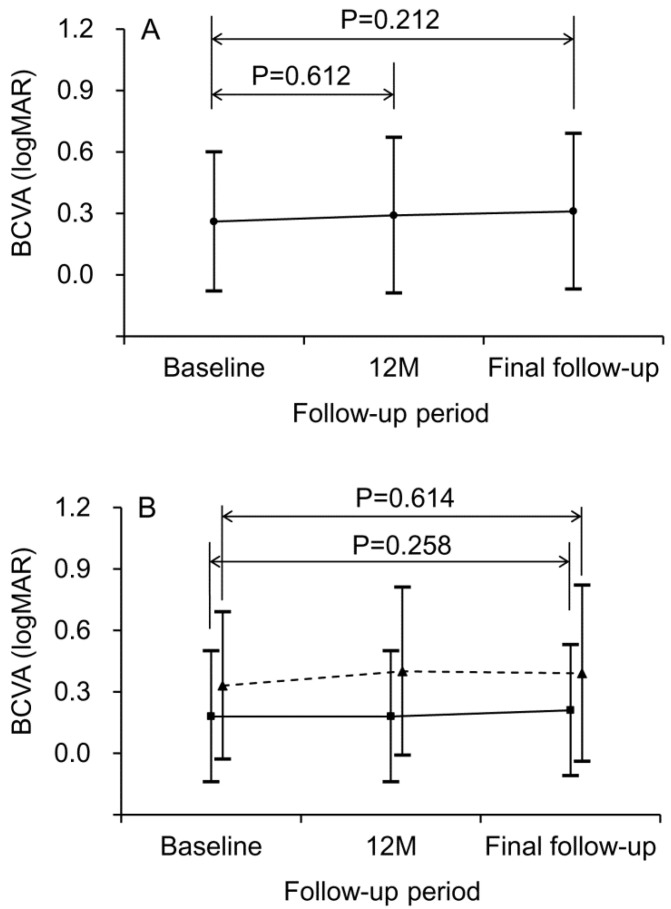
Changes in BCVA during the follow-up period. (**A**) All 19 patients, (**B**) When divided into 2 groups, according to the lesion size. The solid line indicates the small lesion size group and the dashed line indicates the large lesion size group. Statistical analysis was performed using the Wilcoxon signed-rank test with Bonferroni’s correction. BCVA = best-corrected visual acuity, logMAR = logarithm of the minimum angle of resolution, M = months.

The mean logMAR BCVA was 0.26 ± 0.34 at baseline, 0.29 ± 0.38 at 12 months, and 0.31 ± 0.38 at final follow-up (Figure 2A). BCVA at 12 months (*p* = 0.612) and final follow-up (*p* = 0.212) were not significantly different compared to the baseline value. When divided into two groups, according to the lesion size, 9 patients were included in the small lesion size group and 10 in the large lesion size group. In the small lesion size group, the mean logMAR BCVA was 0.18 ± 0.32 at baseline, 0.18 ± 0.32 at 12 months, and 0.21 ± 0.32 at final follow-up (Figure 2B). BCVA at 12 months (*p* = 1.000) and final follow-up (*p* = 0.258) was not significantly different from the baseline value. In the large lesion size group, the mean logMAR BCVA was 0.33 ± 0.36 at baseline, 0.40 ± 0.41 at 12 months, and 0.39 ± 0.43 at final follow-up (Figure 2B). BCVAs at 12 months (*p* = 0.282) and at final follow-up (*p* = 0.614) were not significantly different from the baseline value.

**Figure 3:**
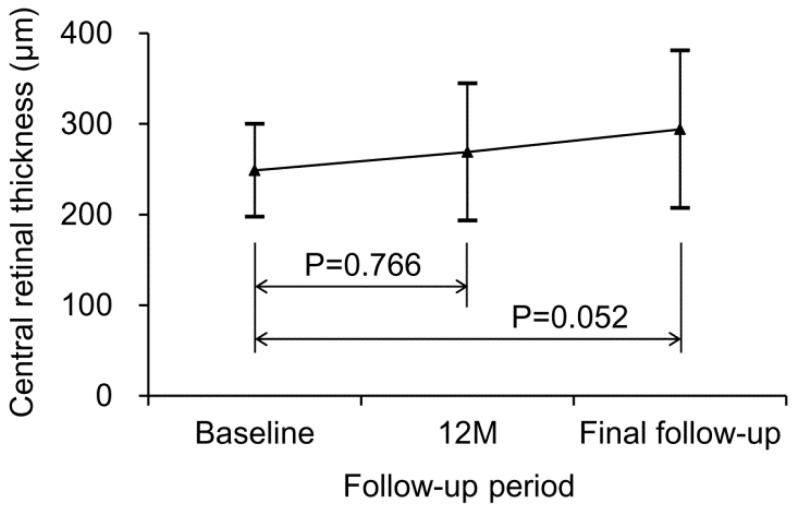
Changes in central retinal thickness (CRT) during the follow-up period. Statistical analysis was performed using the Wilcoxon signed-rank test with Bonferroni’s correction.

During the follow-up period, a deterioration of ≥0.2 logMAR in BCVA was noted in two patients (10.5%). The mean CRT was 248.8 ± 51.3 µm at baseline, 268.9 ± 75.8 µm at 12 months, and 294.1 ± 86.8 µm at final follow-up (Figure 3). CRT values at 12 months (*p* = 0.766) and final follow-up (*p* = 0.052) were not significantly different from the baseline value.

The clinical courses of the two patients who exhibited deterioration of ≥0.2 logMAR in BCVA are described in the following. A 69-year-old man was followed up for 36 months. The patient did not have diabetes mellitus or hypertension, and the lesion size was 8.02 mm^2^. During the follow-up period, the patient received four aflibercept injections. Cataract surgery was additionally performed at 16 months. No other treatment (e.g., PDT) was administered for PCV. The baseline logMAR BCVA was 0.70, which deteriorated to 1.00 at final follow-up. The other, 79-year-old man was followed up for 13 months. The patient had hypertension, and the lesion size was 5.40 mm^2^. During the follow-up period, the patient received two aflibercept injections and one bevacizumab injection. No other treatment was administered. The baseline logMAR BCVA was 0.70, which deteriorated to 1.00 at final follow-up.

Multivariate analysis revealed that lesion size was significantly associated with lesion reactivation (*p* = 0.009; Table 2). A lesion size ≥3.0 mm^2^ was noted among 8 of 10 patients (80.0%) with lesion reactivation, and among 2 of 9 patients (22.2%) without lesion reactivation. However, other factors, including age (*p* = 0.647), sex (*p* = 0.134), BCVA (*p* = 0.388), CRT (*p* = 0.572), number of polypoidal lesions (*p* = 0.657), and serous PED (*p* = 0.301), were not associated with lesion reactivation.

## 4. Discussion

The advent of anti-VEGF therapy has revolutionized neovascular AMD treatment [23]. In early clinical trials, fixed-dose regimens, characterized by continuous injections at 4-to-8-week intervals, were advocated [24,25]. Although fixed-dose regimens showed favorable treatment outcomes, there was an increase in treatment burden due to the high cost of the drug, as well as the necessity for frequent hospital visits, thus warranting the development of more efficient treatment regimens.

An as-needed regimen, which is characterized by administering injections only when the lesion is reactivated, was first developed by Fung et al. to efficiently treat neovascular AMD [19]. In an early clinical trial, an as-needed regimen led to a similar visual outcome with markedly fewer injections compared with a fixed-dose regimen [19]. However, recent studies have suggested that the visual outcome of an as-needed regimen is generally inferior to proactive treatment regimens such as fixed-dose [26] or treat-and-extend [20,27] regimens. Intravitreal anti-VEGF injection imposes a substantial financial and time burden on the patient [28,29] and is frequently associated with pain and anxiety [30,31]. Therefore, due to the patient’s request as well as the doctor’s discretion, it is sometimes impossible to continue the regimen in the long term. Hence, switching to an as-needed regimen can be an alternative treatment option.

To date, limited information regarding the recurrence rate and clinical course after switching to an as-needed regimen is available. In the study by Arendt et al., patients were initially treated with a treat-and-extend regimen [21]. Administration of three consecutive injections, 16 weeks apart, with stable findings, was considered as the “exit criterion,” after which treatment was changed to the as-needed regimen. After switching, 13% of patients had recurrent disease after a mean 37 ± 16 weeks. In addition, PED presence at treatment termination was associated with a high risk of recurrence [21]. Further, the same group reported that the presence of vitreomacular adhesion was associated with a high risk of recurrence after exit from the treat-and-extend regimen [32]. Adrean et al. reported recurrence in 29.4% of patients at a mean of 14 months following cessation of the treat-and-extend regimen [22].

In the present study, we first evaluated the detailed clinical course of PCV patients who were first treated with a fixed-dose regimen followed by an as-needed regimen. The major findings of the present study can be summarized as follows: overall, visual acuity remained stable after switching to the as-needed regimen, with a deterioration of ≥0.2 logMAR in BCVA noted in only 10.5% of patients. Lesion reactivation was noted in 52.6% of patients at a mean of 14.4 ± 9.1 months after the last aflibercept injection. In addition, patients with large lesion size at baseline had a higher risk of lesion reactivation.

The lesion reactivation rate was higher in the present study than in previous studies [21,22]. However, different study protocols and populations were used in all studies. The previous studies stopped the fixed-dose regimen in only a limited proportion of patients who met the exit criteria [21,22]. By contrast, no such criteria were used in the present study, and the treatment was stopped in all patients. Unlike the present study, previous studies included patients with neovascular AMD [21,22]. It has been reported that there are some differences in the reactivation rate between PCV and neovascular AMD after initial loading injections [33], indicating a possible influence of the nature of the disease on the treatment outcome.

It is well established that lesion size is associated with the prognosis of PCV [34,35]. Tsujikawa et al. reported that PCVs with initially larger lesions progressed more rapidly with greater visual deterioration than did those with smaller lesions [34]. In the 3-year follow-up study by Kang et al., larger lesion size was associated with poor visual outcome [35]. In the present study, the lesion reactivation rate in patients with lesions ≥3 mm^2^ was fourfold higher than in those with smaller lesions, suggesting that lesion size is a relevant predictive factor for lesion reactivation.

On the basis of the current results, the following suggestions are made: if fluid and hemorrhage are completely resolved after 1 year of treatment with a fixed-dose regimen, switching to an as-needed regimen can be considered a useful treatment option, especially for patients with small lesion size. In patients with large lesion size, frequent follow-ups are required after switching to an as-needed regimen to avoid delayed detection of lesion reactivation. Continuation of the proactive treatment rather than switching to an as-needed regimen can be considered when preserving vision is essential for a patient’s quality of life (i.e., loss of vision in the fellow eye).

In the present study, the degree of visual deterioration during follow-up was greater in the large lesion size group than in the small lesion size group. However, the BCVA at baseline was not different from that measured at 12 months and at final follow-up in both groups. Considering the small number of patients included in each group, it is possible that the nonsignificant result may have derived from the small sample size. Further studies with larger study populations are required to accurately demonstrate the long-term visual changes based on lesion size.

Anti-VEGF monotherapy is effective in long-term visual preservation in PCV [36]. In the study of Miyamoto et al., ranibizumab monotherapy was found to be superior to PDT [18]. In addition, Wong et al. showed that aflibercept monotherapy was not inferior to aflibercept with rescue PDT [37]. Despite this good efficacy of anti-VEGF monotherapy, it has been demonstrated that PDT and anti-VEGF combination therapy has superior efficacy and efficiency to anti-VEGF monotherapy. Lim et al. showed that the combination of PDT and anti-VEGF therapy resulted in superior visual acuity gain, increased rate of polypoidal lesion regression, and fewer treatment episodes compared with ranibizumab monotherapy [8]. This advantage of combination therapy has also been demonstrated in real-world data. Chong et al. found superior visual acuity gain, a higher proportion of inactive lesions, and quicker time to inactivity following combination therapy, with fewer injections, compared to ranibizumab monotherapy [9]. Doble et al. assessed the incremental cost-effectiveness of combination therapy compared with ranibizumab monotherapy. They found that combination therapy had similar costs but was slightly more effective than ranibizumab monotherapy over a lifetime [38].

In the present study, PDT was not performed throughout the follow-up period. Although previous evidence suggests that combination therapy can achieve superior visual outcomes with fewer anti-VEGF injections [8,9], these studies were based on the outcomes in treatment-naïve patients who underwent combination therapy immediately after diagnosis. Although one study evaluated the efficacy of rescue PDT during anti-VEGF treatment, PDT was performed only in patients who met strict rescue therapy criteria [37]. To date, there is no firm evidence addressing whether performing PDT during anti-VEGF therapy is cost-effective. However, considering the superior cost-effectiveness of combination therapy demonstrated in previous studies [8,9,38], it is possible that the number of anti-VEGF injections can be reduced by administering combination therapy after the first lesion reactivation. Further studies are required to verify this.

The strength of the present study is that we focused on the treatment outcome of switching from a fixed-dose to an as-needed dose regimen in eyes with subretinal hemorrhage secondary to PCV. Considering that submacular hemorrhage is frequently observed on initial presentation in PCV [10], we believe that our results may be useful to clinicians. In addition, our study provides basic information for planning similar studies that can be applied to the entire PCV population.

In addition to its retrospective nature, this study had other obvious limitations. The very small sample size was the primary limitation of the study. Second, there was considerable variation in the follow-up periods. The longest follow-up period (39 months) was three times greater than the shortest (13 months). This difference in follow-up duration among the patients may have influenced the treatment outcomes. Third, the generally recommended strict monthly follow-up when using an as-needed regimen was not conducted. Thus, the timing of lesion reactivation may not have been accurate in some patients. In addition, some of our patients may have been undertreated. Fourth, only patients with submacular hemorrhage were included in the study; hence, the result may not reflect the outcomes of the entire PCV population. Fifth, environmental risk factors such as smoking were not considered. Finally, two different anti-VEGF agents were used, which may have led to skewed results.

In summary, we evaluated treatment outcomes after switching to an as-needed regimen in patients with submacular hemorrhage secondary to PCV who were initially treated with a fixed-dose regimen. During the mean 27-month follow-up period, visual acuity was relatively stable. Lesion reactivation was noted in 52.6% of patients, and large lesion size at baseline was associated with a high risk of lesion reactivation. Considering the small sample size and other limitations of the present study, further controlled studies with larger sample size are required to verify our findings.

## Figures and Tables

**Table 1:** Baseline characteristics of included patients (N = 19).

Characteristic	
Age	67.7 ± 7.0
Sex	
Men	16 (84.2%)
Women	3 (15.8%)
Hypertension	10 (52.6%)
Diabetes mellitus	4 (21.1%)
Best-corrected visual acuity, logMAR	0.26 ± 0.34
Central retinal thickness, μm	248.8 ± 51.3
Serous pigment epithelial detachment	3 (15.8%)
Presence of intraretinal/subretinal fluid or hemorrhage	0

Data are presented as means ± standard deviations or numbers (percentages) as applicable. logMAR = logarithm of the minimum angle of resolution. Baseline characteristics were obtained at 56 weeks after fixed-dose therapy.

**Table 2:** Multivariate analysis of factors associated with lesion reactivation.

Characteristics	*P* *	β	95% CI
Age	0.647		
Sex	0.134		
Best-corrected visual acuity	0.388		
Central retinal thickness	0.572		
Number of polypoidal lesions	0.657		
Size of the lesion	0.009	32.000	2.394–427.744
Serous pigment epithelial detachment	0.455		

* Statistical analysis performed using a binary logistic regression model. CI = confidence interval.
